# Transcriptome dynamics in the asexual cycle of the chordate *Botryllus schlosseri*

**DOI:** 10.1186/s12864-016-2598-1

**Published:** 2016-04-02

**Authors:** Davide Campagna, Fabio Gasparini, Nicola Franchi, Nicola Vitulo, Francesca Ballin, Lucia Manni, Giorgio Valle, Loriano Ballarin

**Affiliations:** CRIBI Biotechnology Centre, University of Padova, Via Ugo Bassi, 58/B, 35131 Padova, Italy; Department of Biology, University of Padova, Via Ugo Bassi, 58/B, 35131 Padova, Italy; Department of Biotechnology, University of Verona, Verona, Italy

**Keywords:** Ascidian, Apoptosis, Blastogenesis, Differentially expressed genes, RNAseq, Tunicata

## Abstract

**Background:**

We performed an analysis of the transcriptome during the blastogenesis of the chordate *Botryllus schlosseri*, focusing in particular on genes involved in cell death by apoptosis. The tunicate *B. schlosseri* is an ascidian forming colonies characterized by the coexistence of three blastogenetic generations: filter-feeding adults, buds on adults, and budlets on buds. Cyclically, adult tissues undergo apoptosis and are progressively resorbed and replaced by their buds originated by asexual reproduction. This is a feature of colonial tunicates, the only known chordates that can reproduce asexually.

**Results:**

Thanks to a newly developed web-based platform (http://botryllus.cribi.unipd.it), we compared the transcriptomes of the mid-cycle, the pre-take-over, and the take-over phases of the colonial blastogenetic cycle. The platform is equipped with programs for comparative analysis and allows to select the statistical stringency. We enriched the genome annotation with 11,337 new genes; 581 transcripts were resolved as complete open reading frames, translated *in silico* into amino acid sequences and then aligned onto the non-redundant sequence database. Significant differentially expressed genes were classified within the gene ontology categories. Among them, we recognized genes involved in apoptosis activation, de-activation, and regulation.

**Conclusions:**

With the current work, we contributed to the improvement of the first released *B. schlosseri* genome assembly and offer an overview of the transcriptome changes during the blastogenetic cycle, showing up- and down-regulated genes. These results are important for the comprehension of the events underlying colony growth and regression, cell proliferation, colony homeostasis, and competition among different generations.

**Electronic supplementary material:**

The online version of this article (doi:10.1186/s12864-016-2598-1) contains supplementary material, which is available to authorized users.

## Background

Metazoans undergo morphogenetic changes, which include embryogenesis (the gradual transition from zygote to larva/juvenile), organogenesis (from organ primordia to their full functionality), regeneration (from wound healing to re-growth of a functional organ), senescence (from full maturity to the progressive loss of organ functionality), and asexual reproduction (from somatic stem cells to an adult). All these changes ultimately rely on modifications of gene expression with the production of different levels of mRNA for housekeeping and luxury proteins as well as regulatory non-coding RNAs. Therefore, variations in RNA expression are of interest, since the analysis of differential gene expression can reveal mechanisms and dynamics at the basis of biological events.

Tunicates are marine filter feeding organisms representing the sister group of vertebrates [1] and the only chordates with species exhibiting asexual reproduction (by budding or blastogenesis) [2]. Ascidians, the major class of tunicates, are sessile animals including both solitary and colonial species [3]. *Botryllus schlosseri* is a cosmopolitan colonial ascidian, commonly found in shallow waters of temperate regions. It reproduces both sexually and asexually, and is now considered a reference chordate for the study of asexual reproduction [4, 5]. It is also considered a good model to analyze: i) the relationship between embryogenesis and blastogenesis, two developmental pathways producing similar individuals (e.g., oozooids and blastozooids) through different reproductive processes (starting from germ cells or somatic stem cells [6]); ii) germ cell recycling and somatic chimeras (reviewed in [7, 8], see also [9]) and somatic cell clearance and turnover [10]; iii) natural apoptosis occurring cyclically in the colony [11, 12] and the mechanisms underlying aging related to tissue regenerative potential and stem cell functionality [13, 14]; iv) the allorecognition phenomenon and its molecular basis [15]; v) the strategies of immune defense [15–17]; vi) the potentiality of the colonial circulatory system as a model for *in vivo* studies of angiogenesis [18, 19]. A draft of the *B. schlosseri* genome has been recently released [4] and its ontology of anatomy and development defined [5].

**Fig. 1:**
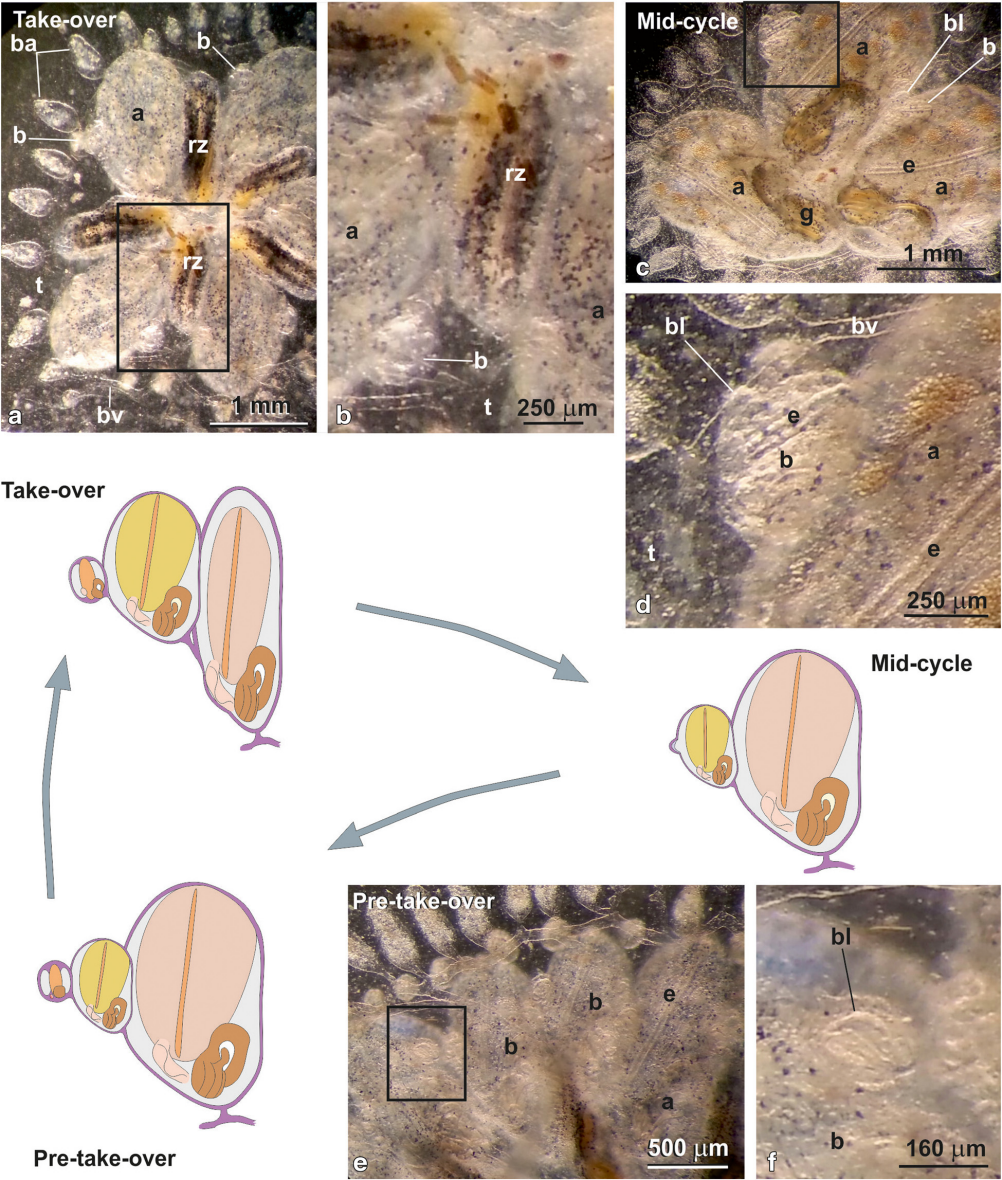
Blastogenetic cycle of *Botryllus schlosseri*. Colonies in take-over (**a**, **b**), mid-cycle (**c**, **d**) and pre-take-over (**e**, **f**) were chosen to study differentially expressed genes. Squared areas in (**a**), (**c**) and (**e**) are enlarged in (**b**), **(d**) and (**f**), respectively. Note that three generations coexist in colonies: filtering adults, buds and budlets. Drawings: branchial basket in pink in adults, pale yellow in buds and dark yellow in budlets; endostyle in orange; epidermis in violet; heart in pale pink; gut in brown. a: adult; b: bud; ba: blood ampulla; bl: budlet; bv: blood vessel; e: endostyle; g: gut; rz: regressing zooid; t: tunic

A colony of *B. schlosseri* (Fig. 1) derives from a single tadpole-like larva, which bears a bud primordium [7, 20]. The larva metamorphoses into a filtering oozooid which develops its bud primordium into an adult zooid (first colonial blastozooid), starting blastogenesis. A colony is formed by many blastozooids, derived by cyclical budding and grouped in star-shaped systems. Usually, a colony contains three blastogenetic generations: filtering adults, buds on adults, and budlets on buds, which develop synchronously [5, 7]. Generation changes, or take-overs (TOs), occurs cyclically (weekly at 20 °C) and defines the blastogenetic cycle, i.e., the interval of time between a generation change and the next [20]. During the TO phase, all adult zooids cease filtering and close their siphons, while their tissues undergo apoptosis and are progressively resorbed. In the meantime, regressing adults are replaced by their buds, which grow to adulthood [20]. The entire lifespan of a zooid, from its appearance as budlet primordium to its resorption at the TO, lasts about 3 weeks at 20 °C.

The colonial blastogenetic cycle is characterized by many morphological changes of zooids, buds and budlets, which, ultimately, imply changes in gene expression. In the attempt to put in evidence these changes, we exploited the next-generation technology of RNAseq and carried out an analysis of the colony transcriptome at various developmental phases. Here, we report on the transcriptome dynamics during the blastogenetic cycle, focusing, in particular, on transcripts of genes involved in cell death by apoptosis.

Fifteen cDNA libraries were sequenced from three key phases (or stages, according to [5]) of the blastogenetic cycle: i) the mid-cycle (MC) phase, more than one day from the preceding or following TO ii) the phase immediately preceding the TO (pre-TO), when the colony is approaching the TO; and iii) the TO phase, when adult zooids are resorbed and replaced by new ones. To analyze differentially expressed genes in these phases, we developed a new bioinformatic tool, in the form of a web-based platform. This platform is available at http://botryllus.cribi.unipd.it where the results of RNA-seq experiments here described are stored and available for further, free gene expression analysis.

## Results and discussion

### Genome annotation enrichment

The first release of *B. schlosseri* genome assembly [4] contains 13 chromosomes and one scaffold that incorporates all contigs not associated with chromosomes (around 30 % of the total). The authors estimated that the *B. schlosseri* genome contains roughly 27,000 genes.

**Fig. 2:**
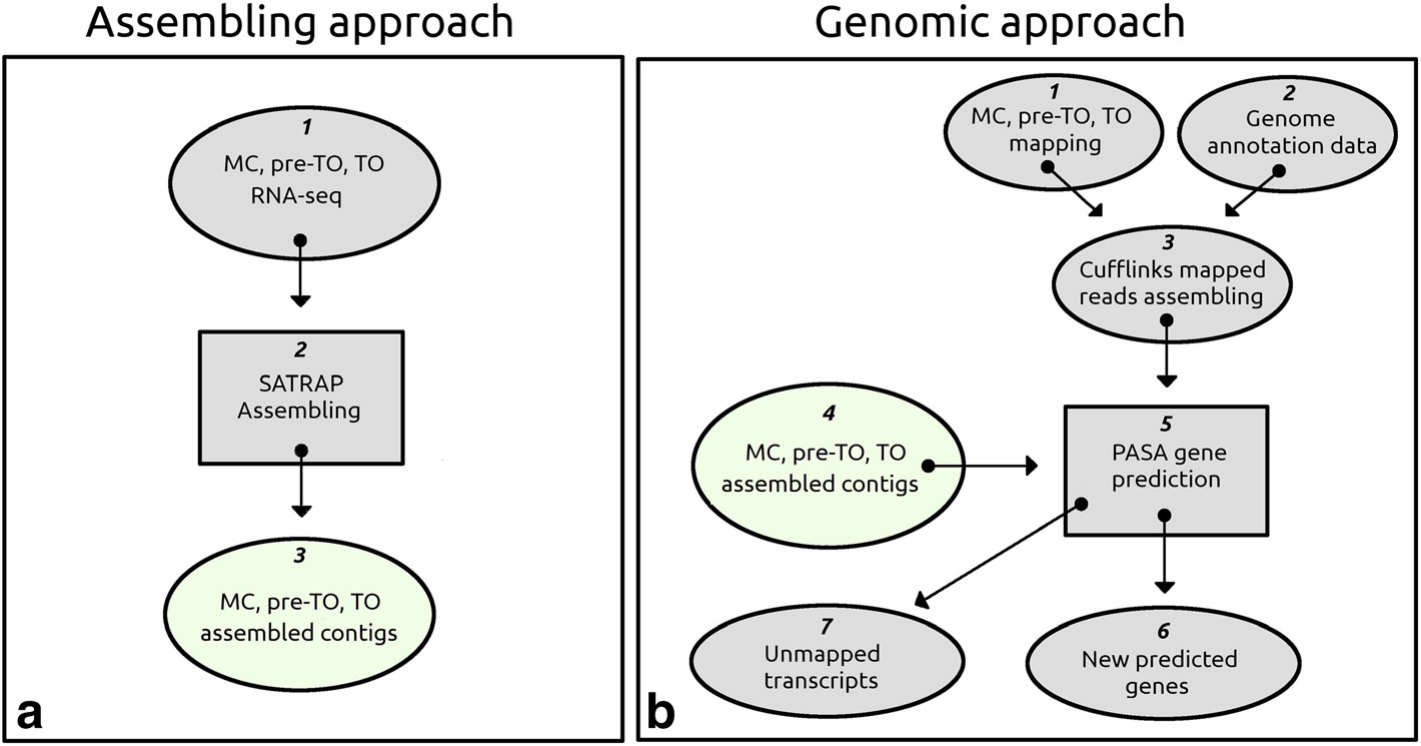
Genome annotation enrichment. **a** (1) the RNA-seq data referred to MC, pre-TO and TO were cleaned for the presence of contaminants (Ribosomal and bacterial sequences) and then (2) assembled and color translated using the program SATRAP. The resulted assemblies (3) contain also the transcripts that did not map in the reference genome because of the lacking of genome information. **b** the mapping information of MC, pre-TO and TO phases (1) as well as the genome annotation data (2) were passed to the program CUFFLINKS (3). The parsimonious dataset of transcripts produced by the program CUFFLINKS and the assembling information of each considered developmental phase (4) (coming from Panel A step 3) were analyzed by the program PASA (5) to produce a new gene prediction consistent with the reference genome sequence (6). Unmapped contigs (7) were reconsidered for further analysis

We enriched the genome annotation using specific transcripts of MC, pre-TO, and TO transcriptomes as described in the Methods section. Two approaches were followed [21]. In the first one, the RNA-seq reads were directly assembled to produce a *de novo* transcriptome assembly (Additional file 1) using the program SATRAP [22] (Fig. 2a). In the second approach, “Align then Assemble” or “Genomic approach” (Fig. 2b and Additional file 2), the color-space reads were mapped onto the *B. schlosseri* reference genome using the program PASS [23].

**Fig. 3:**
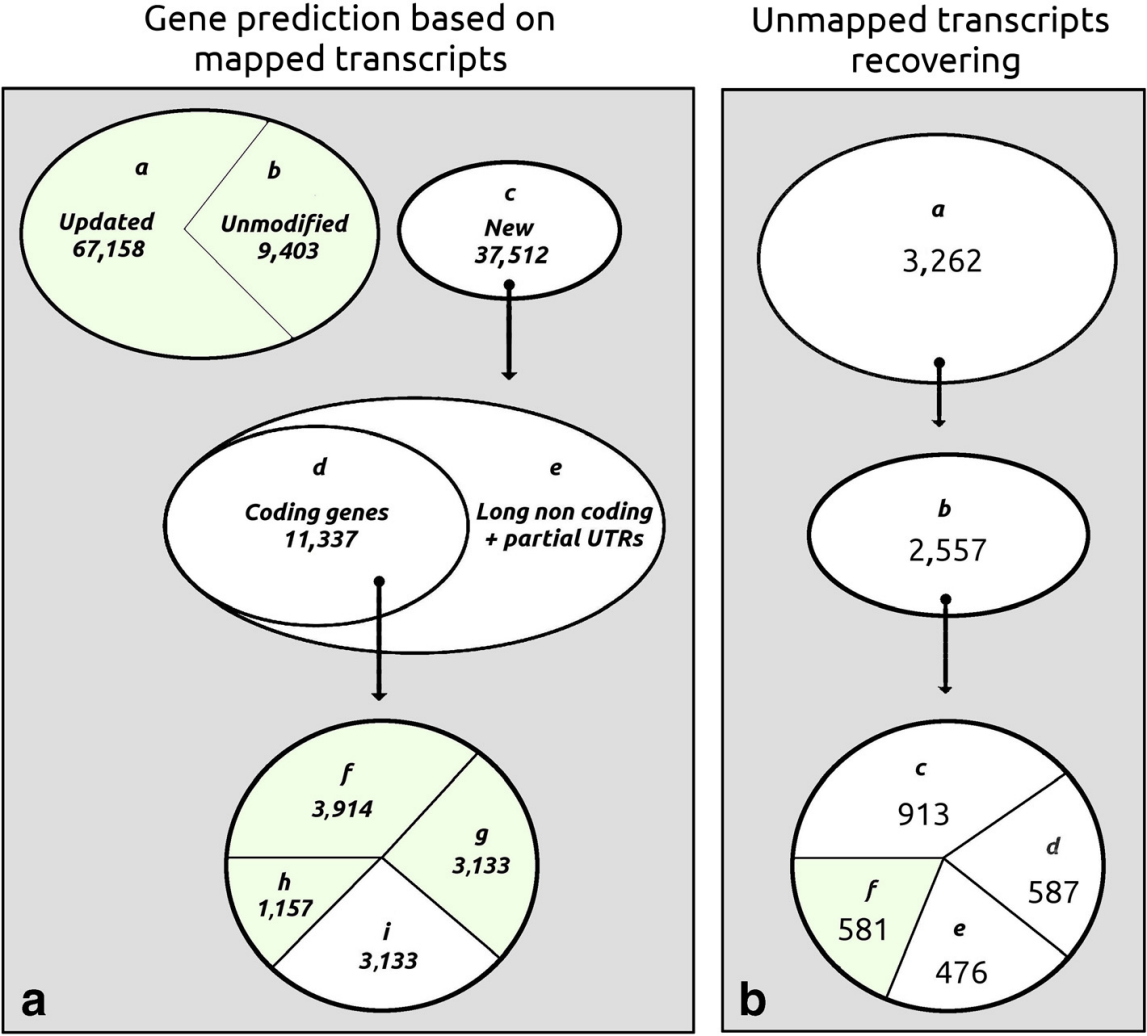
Gene prediction statistics. Statistics inferred using the mapped transcripts are shown in (**a**), while statistics inferred using the unmapped contigs are evidenced in (**b**). The light green areas indicates the reliable transcripts selected as result of this analysis. **b** the areas (*a*) and (*b*) represent the updated and unmodified gene predictions referred to the genome annotation data. The new gene prediction stressed in (*c*) can be classified into coding genes (*d*) and unreliable information such as long non-coding genes and partial UTRs (*e*). About 62.2 % of coding genes (*d*) consisting of (*f*) and (*g*) were significantly similar to a sequence stored into the non-redundant database; while a percentage of 37.8 % of (*d*) consisting of (*g*) and (*h*) had significant long ORF predictions. (*i*) represents the number of unreliable coding genes discarded, while (*a*), (*b*), (*f*), (*g*) and (*h*) represent the gene predictions considered in the gene expression analysis. **b** the area (*a*) represents the number of transcripts (3262) that mapped less than 10 % of their sequence length onto the reference genome. (*b*) 2557 out of 3262 transcripts evidenced a significant coding potential, (*c*) 913 transcripts had ORFs open at 5′-end; (*d*) 587 transcripts resulted in ORFs open at both ends; (*e*) 476 transcripts resulted in ORFs open at 3′-end, and (*f*) 581 resulted complete ORFs

By considering the regions covered by RNA-seq reads, we were able to update the prediction of most genes of the Voskoboynik’s gene prediction. Taking into consideration that some genes showed alternative splicing isoforms, 67,158 transcripts were updated. Newly predicted transcripts, absent in the old annotation, amounted to 37,512; 11,337 of these resulted coding genes, while the remaining seem to be transcribed into non-coding RNAs. Results obtained from the gene prediction analysis are shown in Fig. 3a and further information are included in the Additional file 3.

Unreliable transcripts, not consistent with the genome sequence were discarded, but those resulting entirely unmapped (3262) were reconsidered for further investigations (Fig. 3b and Additional file 4).

**Fig. 4:**
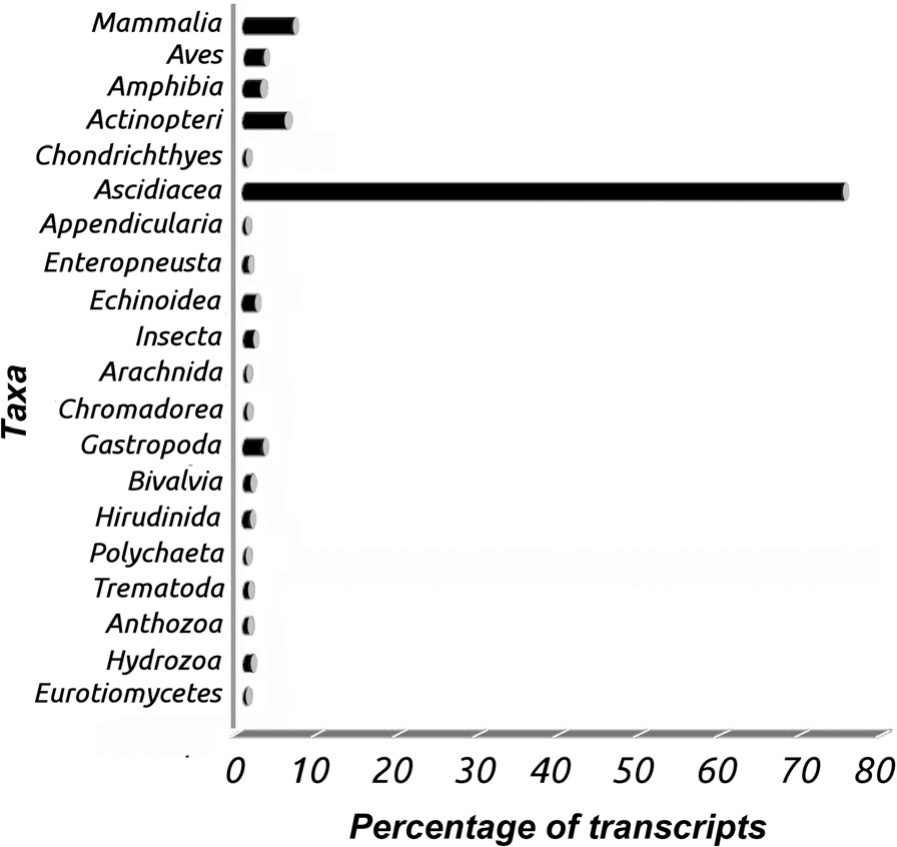
Taxonomic analysis of 581 ORFs. This graph represents the proportion of unmapped transcripts that have inherited the taxonomic classification by similar and significant proteins (400). As expected, the taxon of Ascidiacea resulted the more represented one

The assembled transcripts with an open reading frame (ORF) that had a significant coding potential were 2557, out of 3262 mentioned above [24]. More precisely, 581 transcripts were resolved as complete ORFs, 913 transcripts were resolved as ORFs open at 5′-end, 476 transcripts were resolved as ORFs open at 3′-end, and 587 transcripts resulted in ORFs open at both ends. The complete ORFs were analyzed to minimize the inclusion of putative chimeric assemblies and quantifying the presence of contaminants. To accomplish this purpose the 581 complete ORFs were translated in silico into amino acid sequences and then aligned onto the non-redundant protein sequence database using the program BLASTP [25]. The same ORFs were also aligned onto the nt database (Partially non-redundant nucleotide sequences) using the TBLASTN program [26]. All mapped transcripts were classified according to the taxonomy inherited by similar and significant alignments (Fig. 4) that is also reported in Table 1.

**Table 1:** Taxonomic analysis of 581 ORFs

TAXON	BLASTP	TBLASTN
Mammalia	24	22
Aves	9	11
Amphibia	9	9
Actinopteri	21	24
Chondrichthyes	1	4
Ascidiacea	297	303
Appendicularia	1	0
Enteropneusta	2	9
Echinoidea	6	5
Insecta	4	5
Arachnida	1	1
Chromadorea	1	1
Gastropoda	9	3
Bivalvia	3	0
Hirudinida	3	3
Polychaeta	1	1
Trematoda	2	1
Anthozoa	2	3
Hydrozoa	3	1
Eurotiomycetes	1	1
Total	400	417

Proportion of unmapped transcripts, which inherited the taxonomic classification by similar and significant alignments, resulted from BLASTP and TBLASTN mapping onto non redundant sequence database (nr) and partially non redundant nucleotide database (nt). The e-value was set to 10-5, whereas the percentage of sequence identity was set to the default value. As expected, the taxon of Ascidiacea resulted the most represented

As expected, the class Ascidiacea is the most represented, including about 74 % of the transcripts. The finding that some ORFs have their most similar counterpart in organisms different from Ascidiacea, is of interest and raises the possibility that they could be contaminants from coexisting organisms. Although we cannot exclude that some of these sequences may be contaminants, it should be considered that the *B. schlosseri* colonies were grown in the laboratory under controlled feeding conditions. Furthermore, it is noticeable that more than 15 % of the non-Ascidiacea sequences belong to other chordate vertebrates, and in particular to mammals, which are much better known at the genomic level. On the basis of these considerations, the 581 transcripts were added to those obtained from the gene prediction analysis and the entire dataset was analyzed in the gene annotation process.

**Fig. 5:**
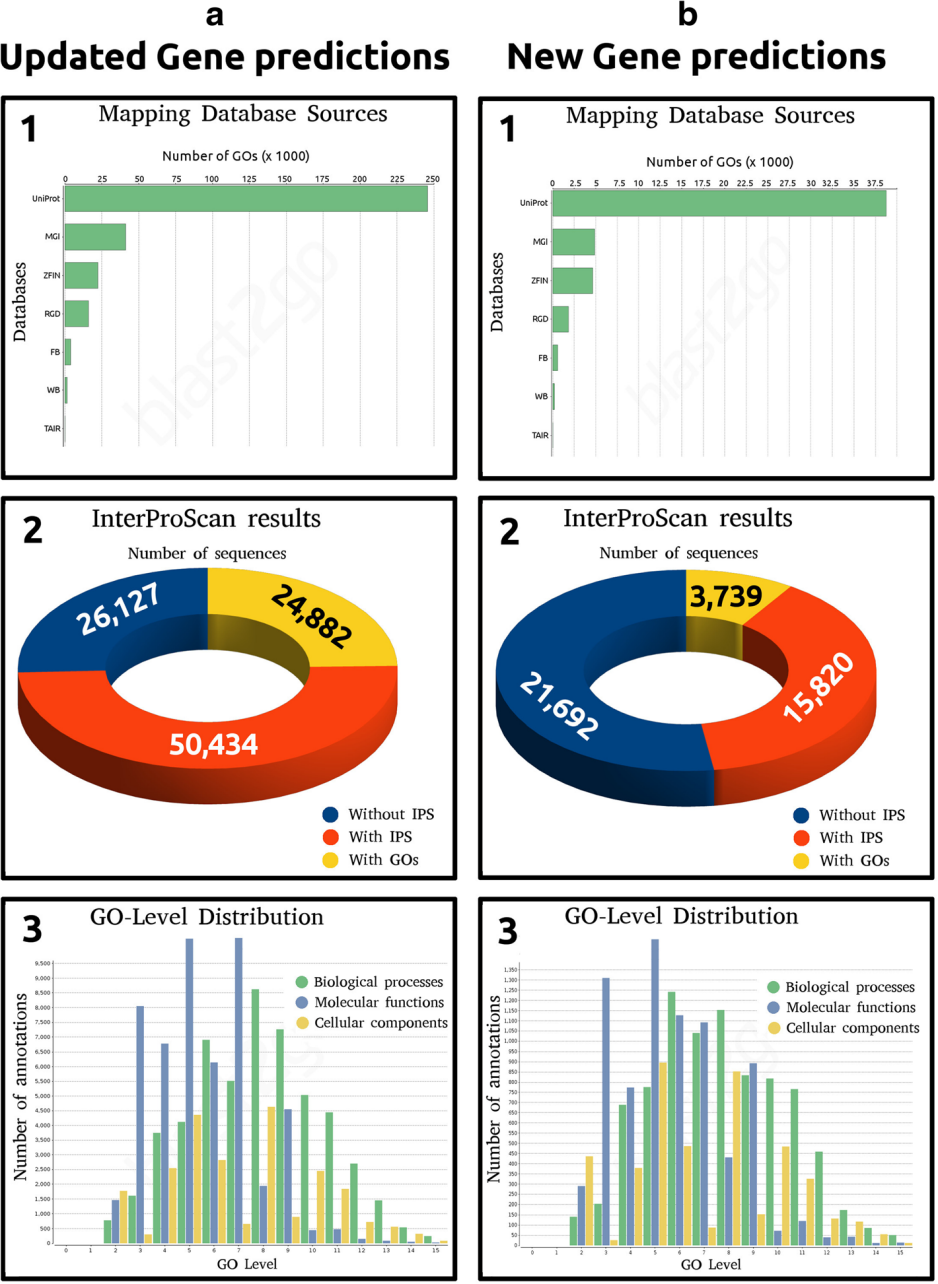
BLAST2GO gene annotation. The transcripts resulted from both genomic and assembling approaches (Fig. 2) allowed updating the old gene predictions (**a**) and producing new gene predictions (**b**). Figures a1 and b1 show the statistics referred to the number of GOs per database source as results of the BLAST similarity searching. Figures a2 and b2 show the number of sequences containing InterProScan (IPS) and GOs given after the integration of data coming from the IPS analysis. Figures a3 and b3 show the GO-Level distribution, respectively for *Biological Processes* (Green), *Molecular functions* (Blue) and *Cellular components* (Yellow)

All transcripts were annotated using the Blast2GO annotation procedure [27]. Updated and new gene predictions were considered as two different datasets and Fig. 5 shows some results of statistical analyses. The majority of annotations come from the UniProt Knowledgebase database source (Fig. 5a1 and b2). A consistent fraction of IPS (protein motif resulted from the InterProScan analysis) found for both datasets resulted associated with Gene ontology (GO) terms (Fig. 5a2 and b2). The total number of annotations, referred to updated gene predictions, was 129,275 while 20,896 annotations were associated with new gene predictions. The mean GO-level resulted 6.76 and 6.74 for both analyses (Fig. 5a3 and b3).

### A web interface for comparative analysis of *B. schlosseri* transcriptomes

A web-based platform was developed to investigate gene regulation in the considered blastogenetic phases. The system includes several programs, mainly devoted to the comparative analysis of transcriptomes, and a web-based interface, which allows intersecting all the information resulting from specific queries. Both queries and statistical functions make possible an overview of the genetic changes under different developmental stages. Genes grouped into GO categories were compared to highlight the changes in transcription and focus the main information. The web-based platform and all scientific data are available without restrictions at web site http://botryllus.cribi.unipd.it/.

### The blastogenetic cycle: an overview of genetic changes

**Fig. 6:**
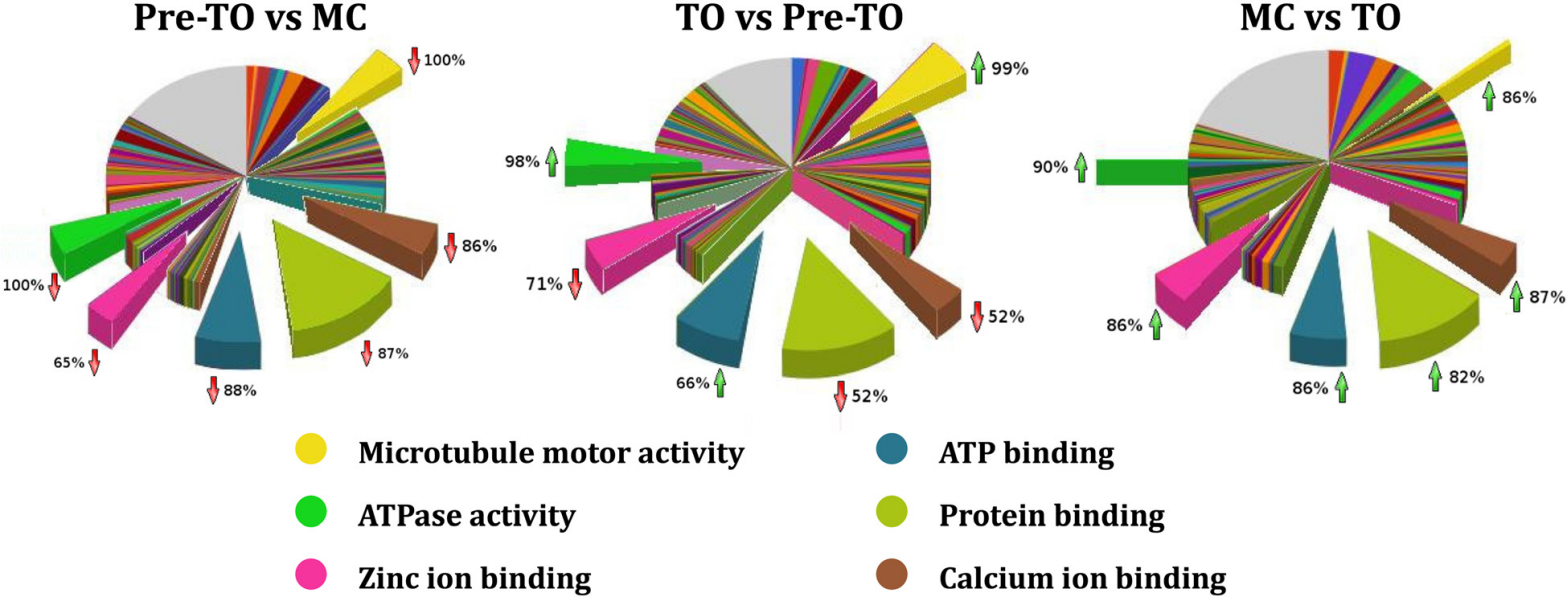
Overview of the differentially expressed genes grouped into the GO domain *Molecular functions*. Differentially expressed genes obtained from each couple of analyzed conditions were grouped into GO definitions (slices) and then compared to analyze the differences of the most represented ones. In the comparison MC *vs* TO, the functional definitions *Microtubule motor activity* and *ATPase activity* resulted strongly reduced in the total number of involved genes as evidenced by the thin slices in yellow and green. The percentage of genes resulted up or down-regulated (green or red arrows) for a specific GO definition is indicated nearby to each slice

A statistical analysis was performed according to the method proposed by Wang and collaborators [28]. Mainly, it integrates the Fisher’s exact test [29] and the likelihood ratio test [30]. We considered the following comparisons: pre-TO *vs* MC; TO *vs* pre-TO, and MC *vs* TO. Significant differentially expressed genes obtained from each analysis were classified within the GO definitions and each category was compared with those coming from other analyses. In order to evaluate the differences in terms of gene number and differential expression, genes belonging to the same GO definition were compared quantitatively. Those more represented, in terms of gene number, related to the ontology domain *Molecular functions* and are reported in Fig. 6.

While the number of differentially expressed genes included in the GO definitions *Calcium ion binding*, *Protein binding*, *ATP binding* and *Zinc ion binding* resulted almost similar in all the compared conditions, a consistent number of differentially expressed genes included in the *Microtubule motor activity* and *ATPase activity* drastically decreased when comparing MC with TO. As shown in Fig. 6, these genes changed their expression also when comparing pre-TO with MC and TO with pre-TO, and related genes involved in myosin and dynein complex formation showed the same behavior (Table 2). Precisely, 39 genes involved in myosin-, dynein- and axonemal dynein-complexes appeared under-expressed in the pre-TO compared with MC, while 62 genes resulted over-expressed in the TO phase. In agreement with this evidence, the majority of genes involved in the ATPase activity showed similar behavior. These data fit the hypothesis that the ATP/ADP hydrolysis is essential for the microtubule activity required for the resorption of the adult zooids, the growth of the buds to adulthood and to complete the morphogenesis of budlets which become buds.

**Table 2:** Differentially expressed genes involved in *Microtubule motor activity*

GO code	GO definition	Down/Up regulated		
		pre-TO *vs* MC	TO *vs* pre-TO	MC *vs* TO
GO:0016887	ATPase activity	39/0	1/51	1/10
GO:0016459	myosin complex	2/0	1/2	0/2
GO:0030286	dynein complex	16/0	1/27	3/4
GO:0005858	axonemal dynein complex	21/0	0/33	NA

List of the GO definitions related to *Microtubule motor activity* and *ATPase activity* for each couple of analyzed conditions

Motor proteins, exploiting the cytoskeleton for movement, can be classified on the basis of their substrates. Actin motors, such as myosin, move along microfilaments through interaction with actin, whereas microtubule motors, such as dynein and kinesin, move along microtubules through interaction with tubulin. Our evidence indicates that only a few genes, involved in the formation of myosin complexes, changed their expression during the blastogenetic cycle. Conversely, a high number of differentially expressed genes were involved in dynein complexes and microtubule formation, as stressed in Table 2. This evidence suggests that the dynein-based tubulin motors are important in the progression of the blastogenetic cycle, especially when TO is approaching.

**Fig. 7:**
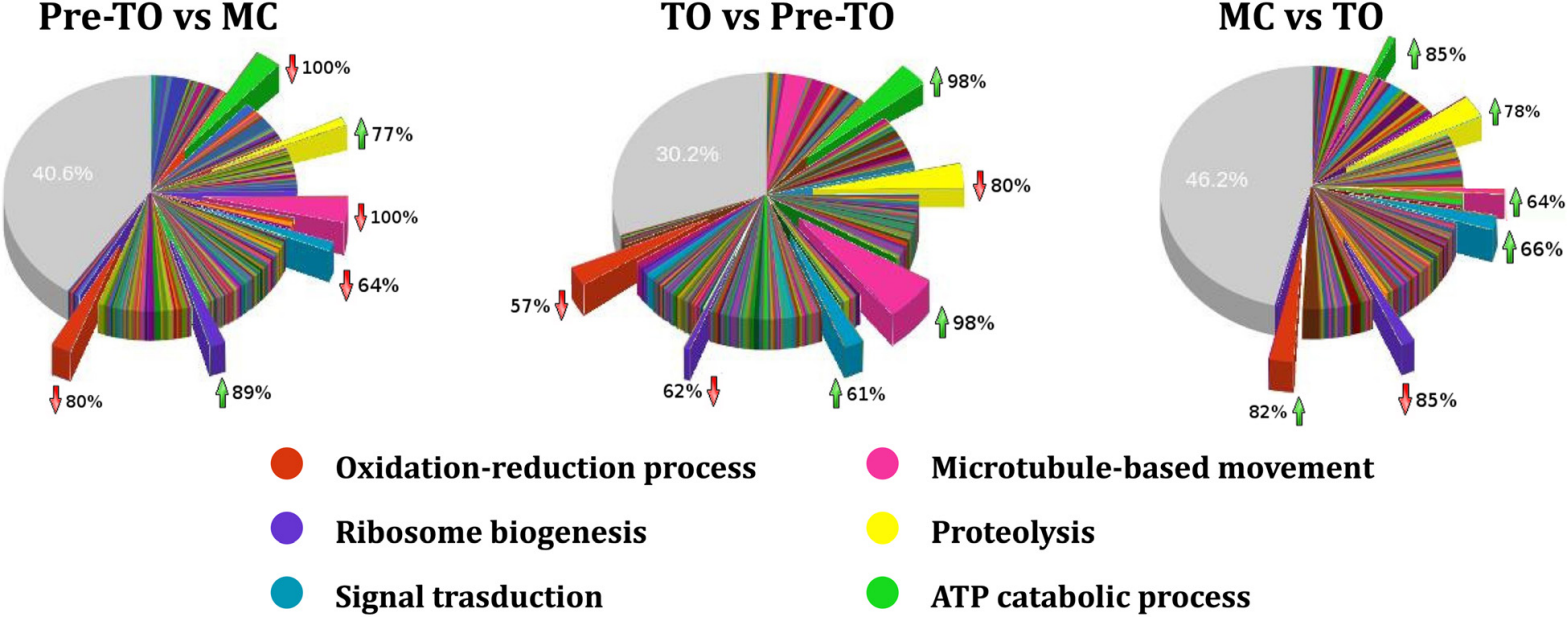
Overview of the differentially expressed genes grouped into the GO category *Biological Processes*. Differentially expressed genes obtained from each couple of considered conditions were grouped into GO definitions (slices) and then compared to analyze the differences of the most represented ones. In the comparison MC *vs* TO, the biological processes *ATP catabolic process* and *Microtubule-based movement* resulted strongly reduced in the total number of involved genes as evidenced by the thin slices in green and pink. The same behavior was found for *Ribosome biogenesis* evidenced by the thin slices in blue in the comparison TO *vs* pre-TO. The percentage of genes up- or down-regulated (green or red arrows) for a specific GO definition is indicated nearby to each slice

As reported in Fig. 7, the analyses of genes involved in *Biological Processes* gave results very similar to those described in Fig. 6. Results concerning the GO definitions *ATP catabolic process* and *Microtubule-based movement* are similar to the previously described data on *Microtubule motor activity* and *ATPase activity*, for both number of genes and regulation trend (see also Table 3).

**Table 3:** Differentially expressed genes involved into the GO category *Biological processes*

GO code	GO definition	Down/Up regulated		
		pre-TO *vs* MC	TO *vs* pre-TO	MC *vs* TO
GO:0001539	ciliary or bacterial-type flagellar motility	21/0	0/33	NA
GO:0006200	ATP catabolic process	32/0	1/41	2/11
GO:0006412	translation	2/14	5/2	22/4
GO:0006508	proteolysis	3/10	36/9	13/45
GO:0007018	microtubule-based movement	39/0	2/79	5/9
GO:0042254	ribosome biogenesis	2/17	5/3	22/6

List of the GO definitions that are more represented for at least one couple of analyzed conditions

The number of differentially expressed genes involved in *Ribosome biogenesis* increased drastically in the comparison of pre-TO with MC (Fig. 7 and Table 3). The other two comparisons (TO *vs* pre-TO and MC *vs* TO) showed that the majority of the differentially expressed genes resulted down-regulated. This supports the hypothesis that the ribosome biogenesis is mainly associated with the final phases of the blastogenetic cycle, probably because of an increased translational activity related to bud growth or the appearance of a new bud generation.

*Proteolysis* is the breakdown of proteins into small peptides or amino acids. Proteolysis serves many purposes: i) breakdown of proteins providing amino acids for basal metabolism and development; ii) cleavage of polypeptide chains for the activation the protein itself; 3) regulation of some physiological and cellular processes and prevention of the accumulation of unwanted or altered proteins inside cells.

The number of differentially expressed genes involved in *Proteolysis* resulted high in the comparisons TO *vs* pre-TO and MC *vs* TO, but decreased in the comparison pre-TO *vs* MC (Table 3, Fig. 7). A detailed summary of the expression of genes involved in proteolysis is shown in Table 4. The majority of the observed proteolytic activities is ascribable to serine-type peptidases (also known as serine proteases or serine endopeptidases) *i.e.*, enzymes cleaving peptide bonds carrying a serine as the nucleophilic amino acid at the active site of the enzyme [31]. In humans, they coordinate various physiological functions, including digestion, immune response, blood coagulation and reproduction [32]. More than 20 serine protease genes resulted activated after the TO. However, the low presence of differentially expressed genes and the high number of read counts in the comparison of pre-TO *vs* MC suggests that there were no changes in the regulation of these enzymes during this developmental transition.

**Table 4:** Differentially expressed genes involved into *Proteolysis*

GO code	GO definition	Down/Up regulated		
		pre-TO *vs* MC	TO *vs* pre-TO	MC *vs* TO
GO:0004252	serine-type endopeptidase activity	0/1	19/1	2/19
GO:0004867	serine-type endopeptidase inhibitor activity	3/0	5/1	0/13
GO:0008236	serine-type peptidase activity	1/0	1/0	0/3
GO:0030414	peptidase inhibitor activity	4/1	3/0	0/5
GO:0004866	endopeptidase inhibitor activity	3/0	2/0	0/3
GO:0008237	metallopeptidase activity	2/1	2/2	1/3
GO:0008234	cysteine-type peptidase activity	0/3	2/4	5/0
GO:0004869	cysteine-type endopeptidase inhibitor activity	0/2	0/5	5/0
GO:0004197	cysteine-type endopeptidase activity	0/3	0/1	0/1

List of the GO definitions related to *Proteolysis* for each couple of analyzed conditions

Cysteine-based proteases play important roles in every aspect of physiology and development. In humans and other animals, they are responsible for pro-hormone processing, MHC II-related immune responses, extracellular matrix remodeling, senescence and apoptosis [33]. Interestingly, the cysteine type peptidase activity is higher during pre-TO and TO and inhibited in the MC (Table 4). This fact suggests that the cysteine-type proteases probably contribute to the recycling process of regressing zooids during the generation change.

In the past, the expression pattern of several genes during the blastogenetic cycle of *B. schlosseri* was analyzed by *in situ* hybridization (ISH). Here, we reconsidered the expression of that genes for which the information on the regions considered for designing ISH probes is available: *cytoplasmic actin-1* (*CA1*), *muscular actin-2* (*MA2*), *troponin T-c* (*TnT-c*), *FoxI*, *Six1/2*, *Six 3/6*, *Eya*, *cadherin* (*Cdh*), *rhamnose-binding lectin* (*RBL*) [34–37]. We checked the presence of these genes in our transcriptomes in order to evaluate if they resulted differentially expressed in MC, pre-TO or TO phases (Table 5). The transcripts were identified using the ISH probe sequences, retrieved from the bibliography, but we were not able to univocally identify the transcripts for *CA1*, *FoxI*, *Eya* and *MA2* genes (Table 5). Probably, some probes were specifically designed for different splicing forms not present in our transcriptome data (an example can be retrieved for th*e E*ya probes in [36].

**Table 5:** Expression analysis of previously studied genes

Gene	References	GenBank IDs (mRNA)	ISH probe (nucleotides)	Best hits (scaffold IDs)	stringency conditions: expression level threshold: 0 for *p*-value: 0.05		
					Filter for minimum clues: 3		
					pre-TO *vs* MC	TO *vs* pre-TO	MC *vs* TO
*CA1*	Degasperi et al., 2009 [34]	FN178501.1	NP	g29085.t1			
				g8425.t1			
				g2746.t1			
*MA2*		FN178503.1	1-1376	g27308.t1			**↓**
				g10356.t1			
*TnT-c*		FN178505.1	100-1103	g6900.t1			
*FoxI*	Gasparini et al., 2013 [36]	HE681860.1	1-1167	TCONS_00000859			
				TCONS_00002496			
*Six1/2*		HE681849.1	1-724	g4758.t1			**↑**
*Six3/6*		HE681854	1-1106	g29556.t1			
*Eya*		HE681857.1	1-517	g23845.t1			
				g5457.t1.1			
*Cdh*	Rosner et al., 2007 [37]	U61755.1	3867–4316	g54100.t1	**↑**		**↓**
*RBL -3*	Franchi et al., 2011 [35]	EU051318.1	19-456	g1465.t1			

Results of the expression analysis (last three columns) using the web-based platform for the genes listed in the first column. **↓** or **↑**: under- or over-expressed, respectively. Absence of arrow in the last three columns indicate that there is no significance in the difference of expression level. Second column: references relative to the ISH studies. Third column: reference the GenBank IDs for the transcripts of which the expression were investigated by ISH. Fourth column: nucleotide region utilized for the ISH probe, and for blast searches in the web-based platform. Fifth column: best hits as identified by blast searches and alignment analyses

Our results indicate that, for most of the transcripts, there is no evidence of differential expression (Table 5). However, transcription factor *Six1/2*, adult muscle-type actin *MA2* and cadherin *Cdh*, resulted differentially expressed. *Cdh* transcript was down-regulated at the MC with respect to the TO, and up-regulated in the pre-TO with respect to the MC. No differences in the expression level of the *MA2* and *Six1/2* transcripts were observed when MC and the pre-TO, as well as the pre-TO and the TO were compared; however, the two genes were differentially expressed when the TO and the MC were compared. The unexpected result regarding *MA2* and *Six1/2* (*i.e.*, their down/up regulation in a phase not balanced by an opposite regulation in another phase) can be explained considering that in this work we focused on three defined phases of blastogenesis. We hypothesize that their expression level is balanced in a blastogenetic phase not considered here.

Unlike *Six1/2*, the transcription factor *Eya* did not result differentially expressed. Considering that the Eya protein is a cofactor of the Six protein [38], and that previous ISH experiments had shown a comparable spatio-temporal pattern of the two transcripts during blastogenesis [36], we explain this discrepancy considering the multiple roles of the two proteins [39] which might be independently regulated in their gene expressions.

*CA1* was not differentially expressed in the three blastogenetic phases. This stable expression level confirms the goodness of the choice of cytoplasmic actin as reference gene for quantitative PCR (polymerase chain reaction) experiments.

### Relative RT-PCR validation

**Fig. 8:**
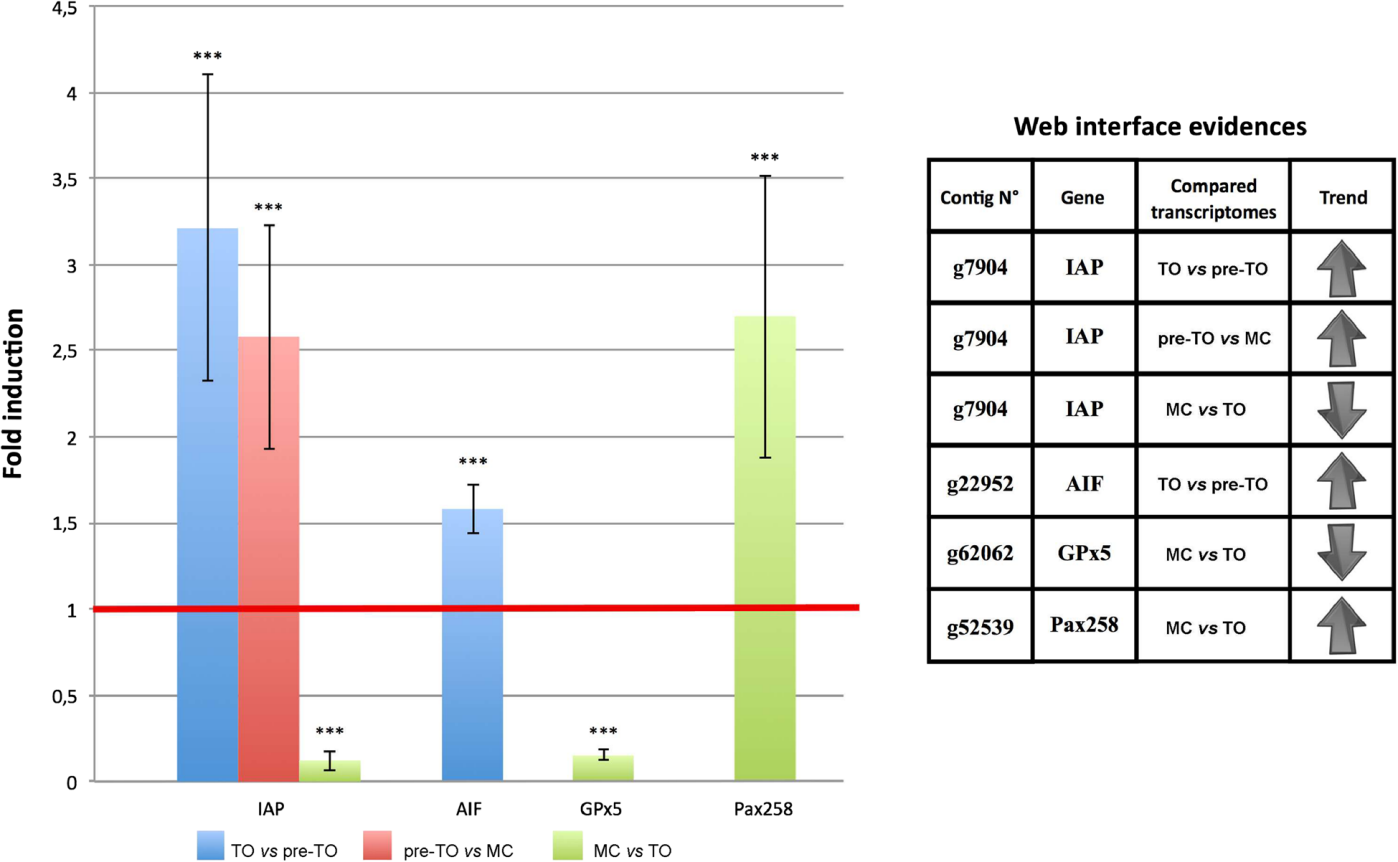
Relative expression levels of *IAP*, *AIF*, *GPx5* and *Pax258* in different phases of the blastogenetic cycle. Each bar of the histogram corresponds to the average of three independent experiments ± SD. Significant differences, with respect to control (set as 1), are marked by asterisks. ***p < 0.001

In order to validate the differential expression for some transcripts, we performed experiments of relative RT-PCR (rRT-PCR) on *inhibitor of apoptosis* (*IAP*), *apoptosis inducing factor* (*AIF*), *glutathione peroxidase 5* (*GPx5*), and *Pax258* transcripts. The level of transcripts for IAP significantly increased during TO with respect to pre-TO, and during pre-TO with respect to MC. Conversely, it decreased in MC with respect to TO. The level of transcripts for AIF increased in TO with respect to pre-TO. The level of transcripts for GPx5 and Pax258 decreased and increased, respectively, in MC with respect to TO. All the results are in agreement with the web interface analyses (Fig. 8).

### *Search for differentially expressed genes in* B. schlosseri*: the case of apoptosis-related genes*

*B. schlosseri* is a useful model organism for studying apoptosis. It offers the advantage of a natural, massive induction of apoptosis during its cyclic generation changes without any external manipulation or drug administration [12, 40, 41]. The diffuse cell death is concentrated in tissues of adult zooids. At the same time, cell proliferation occurs in buds and budlets which grow to adult and bud size, respectively, and in hematopoietic niches to produce new hemocytes entering the circulation and replacing effete circulating cells [12, 42].

Among the differentially expressed transcripts we selected those included in apoptosis-related categories (Table 6). We identified 24 genes that play a role in apoptosis of *B. schlosseri*. Six genes are involved in the apoptosis activation and seven in its inhibition; seven genes take part in apoptosis regulation; the remaining four genes are in relation to apoptosis with other roles, and are not discussed in detail here.

**Table 6:** Apoptosis-related genes obtained comparing transcriptomes

Role	scaffold ID	vertebrate orthologous Gene Abbreviations	Gene name	Pre-TO *vs* MC	TO *vs* Pre-TO	MC *vs* TO
Pro apoptotic	TCONS_00024434	*Pde11a*	*cAMP-GMP Phosphodiesterase 11A*	**↑**		
	g19982.t1	*Gapdh*	*glyceraldeide 3-p dehydrogenase*	**↓**		
	g22952.t1	*Aifm2*	*Apoptosis inducing factor 2*	**↓**	**↑**	
	g8767.t1	*Casp2*	*Caspase 2*	**↑**		
	g6698.t1.1	*Insr*	*Insulin receptor*	**↑**		
	g16248.t18	*Stpg1*	*O-Methylguanine-induced apoptosis 2*		**↑**	
Anti apoptotic	g33523.t1	*Api5*	*Apoptosis inhibitor 5*	**↓**		**↑**
	g4758.t1	*Six1*	*Homeodomain protein Six 1*		**↓**	**↑**
	TCONS_00107696	*PLK1*	*Polo-like Kinase 1*	**↑**		
	g61041.t1	*Ubqln1*	*Ubiquilin 1*		**↓**	
	g40469.t1	*Egfr*	*Epidermal growth factor receptor*	**↓**		
	g43714.t1	*Gclc*	*γ-glutamyl-cysteine ligase catalytic subunit*	**↓**		
	g7904.t1	*Iap*	*Inhibitor of apoptosis*	**↑**	**↑**	**↓**
Regulator	g48839.t1	*Set*	*Template.activating factor 1*			**↑**
	g9434.t1	*Ogt*	*O-N-acetylglucosamine transferase*		**↓**	
	g23050.t1	*Sf3b1*	*Splicing factor 3B, subunit 1*	**↓**	**↓**	**↑**
	g15568.t1	*Scrib*	*Scribble*			**↑**
	g32829.t1.1	*Dynll1*	*Dinein light chain 1*		**↑**	**↓**
	g49777.t1.1	*Cul1*	*Cullin-1*		**↓**	
	g63857.t1	*Cbs*	*cystathionine-beta-synthase*		**↓**	**↑**
Others	g1405.t1.1	*Col3a1*	*collagen, type III, alpha 1*	**↓**		
	g8626.t1	*Aars*	*Alanine-tRNA ligase*			**↑**
	g39299.t1	*Acsl1*	*Long chain fatty acid-CoA ligase 1*	**↓**		
	TCONS_00030959	*Ran*	*GTP-binding nuclear protein ran*			**↑**

Arrows pointing upward means up-regulation, arrows pointing downward means down-regulation. *B. schlosseri* transcripts code are reported in "scaffold ID" column

#### Genes involved in apoptosis activation

In agreement with previous results [12, 41, 43], our data showed that pre-TO and TO are the phases in which major variations of apoptosis-related genes are observable. Our analysis evidenced the following differentially expressed genes: *cAMP-GMP phosphodiesterase*, *glyceraldehyde-3-phosphate dehydrogenase* (*GAPDH*), *apoptosis inducing factor* (*AIF*), *caspase 2*, *insulin receptor* (*IR*) and *O-methylguanine-induced apoptosis 2* (*Mapo2*).

The over-expression, in pre-TO, of *cAMP-GMP phosphodiesterase* indicates that cAMP-mediated signaling pathways are involved in the induction of apoptosis. GAPDH influences the pro-apoptotic mitochondrial membrane permeabilization [44]. Its transcript is under-expressed in pre-TO with respect to MC, probably related to a slow activation of the mitochondrial induction pathway. This fits the observed decrease, passing from MC to pre-TO, of the expression of AIF, a mitochondrial protein responsible for mediating cell death independently from caspases [45]; AIF then increases its expression at TO.

Caspase 2 is an effector caspase able to induce the release of cytochrome c from mitochondria [46] and its over-expression in pre-TO with respect to MC, indicates that mitochondria–related apoptosis really starts at pre-TO.

Results on *IR* and *Mapo2* were unexpected. *IR* over-expression at pre-TO fits the recent description of IR as a dependence receptor [47]. IR mediates apoptosis promoting, by unknown mechanisms, Bax- and caspase 3-mediated cell death [48]. Mapo2 is one of the most important proteins involved in the execution of apoptosis induced by O6-methylguanine [49]. It is activated by mutagenic insults that lead to the formation of O6-methylguanine, when the specific repair protein, O6-methylguanine-DNA methyltransferase (MGMT) fails to transfer the methyl group from O6-methylguanine to a methyl-acceptor cysteine residue [50, 51]. Our results, indicating an over-expression of *Mapo2* during the TO, suggest that DNA alkylation is important in the activation of *B. schlosseri* natural apoptosis*.*

#### Genes involved in apoptosis inhibition

The following genes resulted differentially expressed: *apoptosis inhibitor 5* (*API5*), *homeodomain protein Six*, *polo-like kinase 1* (*PLK1*), *ubiquilin-1*, *epidermal grow factor receptor* (*EGFR*), *γ-glutamyl-cysteine ligase catalytic subunit* (*GCLC*) and *inhibitor of apoptosis* (*IAP*).

*API5* and the *homeodomain protein Six* are over-expressed in MC with respect to TO, in accordance with previous data indicating lower amount of cell death far from the generation change [52]. API5 and the homeodomain protein Six act in a similar way, trough the inactivation of pro-apoptotic protein such as caspases [53–55].

PLK1 is able to bind p53, inhibiting its negative functions on cell cycle [56, 57]. *PLK1* over-expression at pre-TO is probably related to the cell proliferation required for bud to grow to adult size before acquiring functional maturity [20]. *Ubiquilin-1* is under-expressed at TO. In mammals, Ubiquitin-1 has the capability to suppress neuronal cell death [58]; it could play a similar role in *B. schlosseri*, allowing neuronal cell death in regressing zooids. EGFR activates the RAS-MAPK pathway and modulates the induction of apoptosis [59]. In *Drosophila melanogaster*, the down-regulation of an EGFR/Ras/Raf signaling pathway is required for apoptosis [60]. Our data, indicating a reduced transcription of the gene at pre-TO, suggest a similar role of EGFR in *B. schlosseri.*

GCLC is a key enzyme in the synthesis of GSH, which is produced, in ascidians, by a subpopulation of hemocytes [61]. *GCLC* over-expression prevents cell damage and death by oxidative stress. Its under-expression at pre-TO strengthens the proposed role of ROS in the induction of apoptosis at TO [12]. IAP is a negative regulator of caspase activity [62]. Its over-expression at pre-TO and TO can be related to the need to suppress apoptosis before the beginning of a new blastogenetic cycle. The gene expression is switched off in the following MC.

#### Genes involved in apoptosis regulation

Seven genes, important for the regulation of cell death, resulted differentially expressed. They were: *template-activating factor 1* (*SET*), *O-N-acetylglucosamine transferase* (*OGT*), *splicing factor 3B*, *scribble*, *dynein light chain 1*, *cullin-1* and *cystationine ß-synthase*.

SET is a multifunctional protein that exerts a negative regulation of apoptosis induction in mammalian neurons [63]. The increase of *SET* transcription at MC is in accordance with this role.

It is well known that post-translational modifications are important for the modulation of biological events [64]. Johnson and collaborators [65] stressed the role of transfer of O-linked N-acetylglucosamine residues to serines and/or threonines in the regulation of apoptosis. The variation in transcription of the gene for OGT suggests that, also in *B. schlosseri*, the enzyme contributes to the regulation of cell death. Even post transcriptional modifications seem to be important in *B. schlosseri* apoptosis, as indicated by the changes of *splicing factor 3B*. Its over-expression at MC suggests a role of splicing events, similarly to what reported by Laetsch and collaborators [66] in neuroblastoma, in the regulation of the blastogenetic cycle.

It has been shown that deregulation of the polarity protein *Scribble* is involved in the modulation of cell death pathways, in both normal morphogenesis and oncogenesis, acting as a scaffold protein for the activation of Rac signaling pathway [67]. The over-expression, in MC, of a *Scribble* homolog suggests the presence of a similar regulation also in *B. schlosseri.*

Dynein light chain 1 is implicated in the regulation of germ cell apoptosis in *Caenorhabditis elegans* [68]. Our data indicate a variation of the expression of *dynein light chain 1* gene and point to its involvement in *B. schlosseri* cell death*.*

In mammals, cullin-1 is involved in the regulation of neuronal apoptosis [69] whereas cystationine ß-synthase regulates LPS-induced apoptosis in hepatic cells [70]. Homologous genes for cullin-1 and cystationine ß-synthase are present in our transcriptomes and the variation of their transcription supports the hypothesis of their involvement in apoptosis regulation during the *B. schlosseri* blastogenetic cycle.

## Conclusions

The blastogenetic cycle of *B. schlosseri* is a fascinating process, which re-starts every week under laboratory conditions, allowing the cyclical rejuvenation of the colonies and giving the organism a potential never-ending life. In fact, in this animal, birth, development and regression of zooids are continuous, for the contemporary presence, in the colony, of the three blastogenetic generations.

The results reported in this paper contribute to the improvement of the annotation of the first released genome assembly [4].

Moreover, the analyses of differentially expressed genes in the chosen phases of the blastogenetic cycle give an overview of the transcriptional changes occurring during blastogenesis. Recently, a complementary work has been performed [71], where differential expression was investigated comparing fertile and infertile colonies. Both studies paves the way to further investigations of biological processes such as growth and regression, cell proliferation, colony homeostasis, and competition among different generations in the colony.

The case of apoptosis, which we chose as an example, showed candidate genes involved in activation, inhibition and regulation of cell death in specific phases of the blastogenetic cycle. Many of these genes were not investigated previously in *B. schlosseri.* Their study will allow a better comprehension of the role and the importance of apoptosis in colony homeostasis.

## Methods

### Animal collection and RNA extraction

Five colonies of *B. schlosseri* with different genotypes, originally collected from the Lagoon of Venice in the period from September to November 2011, were kept, attached to glass slides, in a large tank with circulating seawater, at the Marine Station of the Department of Biology, University of Padova, in Chioggia. Before their use, colonies were brought to the Department of Biology, on January 2012, where they were reared in standard laboratory condition [72, 73] at 6 °C, a temperature close to the mean winter Lagoon temperature.

Each colony was subdivided in three subclones and each subclone was exploited when they were sexually immature (without mature gonads) at the following colonial developmental phases: MC, pre-TO and TO. According to the staging method proposed by Sabbadin [72], these phases corresponded to 9/8/2, 9/8/5 and 11^2^/8/6, respectively (see also [5, 20]). The subclones were used to prepare fifteen cDNA libraries, using the Applied Biosystems SOLiD™ 5500.

Each RNA extraction was obtained from single subclones, ranging from 110 to 350 mg of wet weight, grinded for 2 min with a frosted glass pestle in a 15 ml tube filled with 10 μl/mg of heated (65 °C) extraction buffer composed of CTAB Lysis buffer (Applichem, cat. A4150) and of 2 % β-mercaptoethanol. Samples were then maintained for 1.5 h at 65 °C in a water bath, shaking them for few seconds every 20 min, and cooled for 2 min on ice. Then, an equal volume of chloroform-isoamyl alcohol (24:1) was added and samples were vigorously shaken until an emulsion was formed. They were then centrifuged for 15 min at 1600 g, at 4 °C. The aqueous phase was collected in 1.5 ml tubes and the SV Total RNA Isolation System (Promega, cat. Z3100) spin protocol was then followed to isolate and concentrate the total RNA. The Invitrogen Qubit Fluorometer and the Thermo Scientific Nanodrop ND-1000 Spectrophotometer were used to check RNA purity and concentration. Ethidium bromide-stained agarose gel (1 %) and the Agilent 2100 Bioanalyzer were used to determine RNA size and integrity.

### Sequencing

The mRNA samples were extracted from total RNA using the Dynabeads® mRNA DIRECT™ Kit (Life Technologies PN 61011). Libraries were prepared for ligation according to the protocol provided by the SOLiD whole transcriptome library kit (Life Technologies, SOLiD™ Total RNA-Seq Kit, PN 4452437). Briefly, samples were purified with the RiboMinus Concentration Module (Life Technologies, PureLink® RNA Micro Scale Kit, PN 12183016), subjected to RNase III digestion for 10 min, retro-transcribed, size-selected by AMPure XP beads and barcoded during final amplification. Raw data has been deposited on NCBI [NCBI:SRR2656922, NCBI:SRR2657206, NCBI:SRR2657210] within the project ID PRJNA298123.

### Gene prediction

All mapping information were analyzed using the program CUFFLINKS [74], in order to produce a parsimonious set of transcripts belonging the phases MC, pre-TO and TO. The assembling information and the generated dataset of transcripts were passed as input to the program PASA [75] to perform a new gene prediction.

The PASA program reports both reliable and unreliable transcripts based on the percentage of mapped sequence size onto the reference genome using a default pre-set stringency. The transcripts that did not map almost entirely (90 % of their sequence length and at least 95 % of their sequence identity) were not considered in the gene prediction analysis because they were unreliable information.

Conversely, all mapped transcripts that had less than 10 % of mapped sequence length onto the reference genome (3262) were recovered for further analysis and those accomplished all the criteria required by the strategy (see the paragraph “Genome annotation enrichment” in the Results and Discussion section) were reconsidered as well as the reliable gene predictions.

### Transcripts recovering

The transcripts resulting unmapped with less than 10 % of their sequence length onto the reference genome were further analyzed using the program Transdecoder from the Trinity package [76]. Subsequently, the transcripts with a significant coding potential and for which a complete ORF was found, were translated in amino acid sequences and then aligned onto the non-redundant sequence database using the program BLASTP.

Next, referring to the similarity information, only the assembled transcripts, which, once translated in silico, showed highly similarity with an annotated protein (best hit), were considered in the subsequent gene annotation process.

### Blast2GO annotation

The annotation was produced using the Blast2GO annotation procedure. Transcripts similar to sequences contained into the non-redundant sequence database were identified using the program Blastx (e-value 10^-4^). Then, protein motifs of predicted transcripts significantly similar to those ones stored into the PROSITE database [77] were identified using the InterProScan [78] program.

All IPSIDs were mapped on GO terms and merged with blast-derived GO annotations to provide one integrated annotation result.

Finally, all transcripts were annotated and all annotations were stored into an internal database ready for the subsequent analyses.

### Quantification of transcript abundance

In order to quantify the abundance of transcriptional variants we used the RSEM program [79]. The SOLiD RNA-seq reads were mapped onto the assembled transcripts using the program PASS. The resulted alignments as well as the gene prediction information were passed to RSEM for the quantification of transcripts abundance.

### Gene expression analysis

Statistical analysis was performed according to [28]. We focused on three different comparison: pre-TO *vs* MC; TO *vs* pre-TO, and MC *vs* TO. This choice was imposed by the interest to verify how many genes were expressed passing from: i) MC to pre-TO (the period featured by budlet morphogenesis, and differentiation of the branchial basket, neural complex and gut, as well as gonad maturation in buds); ii) from pre-TO to TO (when budlets complete morphogenesis, cyto-differentiation occurs in buds, and adults begin TO); iii) from TO to MC (when generation change is completed and new budlets appear) [5, 20].

The statistical test was performed using a *p*-value of 0.05. The differentially expressed genes, confirmed by at least 3 out of 5 biological replicas, were grouped into GO categories. Within each GO category, transcripts from subclones at a specific developmental phase were compared with transcripts from the other two phases.

Since, in order to recognize changes in the transcription regulation during the blastogenetic cycle, we compared pairs of colonial developmental phases in their temporal succession, Eq. 1 and 2 quantify the changes for each GO category, in gene regulation (1) and gene number (2), giving a result ranging between -1 and 1.

1 $$ GRV=\frac{\left[\left(D1-D2\right)+\left(U2-U1\right)\right]}{\left[D1+D2+U1+U2\right]} $$

2 $$ GNV=\frac{\left[\left(D2+U2\right)-\left(D1+U1\right)\right]}{\left[D1+D2+U1+U2\right]} $$

where GRV and GNV indicate the gene regulation variation and the gene number variation, respectively, D1 and D2 represent the number of down-regulated genes found in two considered subsequent blastogenetic phases, and U1 and U2 represent the number of up-regulated genes for the same analyses.

The most represented GO categories, for which GRV and GNV > |0.9|, were selected and reported in the “Result and Discussion” section.

#### Relative RT-PCR

To estimate the level of transcripts for IAP, AIF, GPx5 and Pax258, we used the relative RT-PCR (rRT-PCR) analyses with the SYBR green method (FastStart Universal SYBR Green Master-ROx, Roche). Total mRNA was extracted from *B. schlosseri* colonies at MC, pre-TO, and TO with the SV Total RNA Isolation System (Promega); its purity was determined by the A_260/280_ and A_260/230_ ratio. RNA integrity was determined by visualization of rRNAs in ethidium bromide-stained agarose gels (1,5 %). The first strand of cDNA was reverse-transcribed from 1 μg of total RNA at 42 °C for 1 h in a 20 μl reaction mixture containing 1 μl of ImPromII Reverse Transcriptase (Promega) and 0.5 μg oligo(dT)-Anchor primer or random primers (Table 7). Forward and reverse primers for *IAP*, *AIF*, *GPx5*, *Pax258* and *CA1*, the latter used as housekeeping gene, were synthesized by Sigma Aldrich (Table 7). rRT-PCR analyses were performed using Applied Biosystems 7900 HT Fast Real-Time PCR System. Real time-PCR reactions were performed with the following cycling parameters: 95 °C for 10 min and then 40 cycles of 95 °C for 10 s and 60 °C for 1 min. Each set of sample was run three times and each plate contained cDNA from three different biological samples and negative controls. The method of 2^-ΔΔCt^ [80] was used to estimate gene transcription level. For the statistical analysis, each experiment was replicated at least three times (*n* = 3) with three independent samples; data are expressed as fold induction ± SD. The results were compared with the Student’s *t*-test.

**Table 7:** Primers used for relative RT-PCR experiments

rRT-PCR primers (5′–3′)	
CA1 forward	ACTGGGACGACATGGAGAAG
CA1 reverse	GCTTCTGTGAGGAGGACAGG
IAP forward	GACGAAACCAACGCAGAAC
IAP reverse	GCAGGTCAGCGTTTCTTG
AIF forward	GGTGAGCAAGGGCATAGAAC
AIF reverse	TGAACGACTCCGTGTTTGG
GPx5 forward	GGAAATGGATGGACGCCGCA
GPx5 reverse	CCTAACTCTTCGGTGTATGCGGGAC
Pax258 forward	GCCGAAAGTGGTGGATAAAA
Pax258 reverse	ATTGAACTGACGCTGGGAAC
dT anchor	ACCACGCGTATCGATGTCG(dT)^16^

## Availability of data and materials

Raw data from SOLiD sequencing has been deposited on NCBI [NCBI:SRR2656922, NCBI:SRR2657206, NCBI:SRR2657210] within the project ID PRJNA298123. Data related to gene expression analyses are available without restrictions at web site http://botryllus.cribi.unipd.it/. This web-based interface allows intersecting all the information resulting from specific queries by several programs, which are mainly devoted to the comparative analysis of transcriptomes.

## Additional files

Additional file 1: Assembling approach. Setting of programs used for *de novo* transcriptome assembly. (PDF 78 kb) Additional file 2: Genomic approach. Setting of programs used for genomic approach. (PDF 80 kb) Additional file 3: Gene prediction based on mapped transcripts. Setting of programs used for the gene prediction. (PDF 76 kb) Additional file 4: Unmapped transcripts recovering. Method description and statistics for unmapped transcripts recovering. (PDF 109 kb)

## Abbreviations

AIF: apoptosis-inducing factor
API5: apoptosis inhibitor 5
CA1: cytoplasmic actin-1
Cdh: cadherin
EGFR: epidermal grow factor receptor
GCLC: γ-glutamyl-cysteine ligase catalytic subunit
GAPDH: glyceraldehyde-3-phosphate dehydrogenase
GO: gene ontology
IAP: inhibitor of apoptosis
IPS: InterProScan
IR: insulin receptor
ISH: *in situ* hybridization
MA2: muscular actin-2
Mapo2: O-methylguanine-induced apoptosis
MC: mid-cycle
MGMT: O6-methylguanine-DNA methyltransferase
OGT: O-N-acetylglucosamine transferase
ORF: open reading frame
PCR: polymerase chain reaction
PLK1: polo-like kinase 1
Pre-TO: pre-take-over
RBL-3: rhamnose-binding lectin-3
ROS: Reactive oxygen species
SET: template-activating factor 1
TnT-c: troponin T-c
TO: take-over
