# Supplementary material for: Transcriptome dynamics in the asexual cycle of the chordate Botryllus schlosseri
Source: BMC Genomics. 2016 Apr 2;17:275. doi: 10.1186/s12864-016-2598-1 (PMC4818882; doi:10.1186/s12864-016-2598-1)
Supplement: Additional file 1: — Assembling approach. Setting of programs used for de novo transcriptome assembly. (PDF 78 kb) [file 12864_2016_2598_MOESM1_ESM.pdf]

Assembling approach

SOLiD Reads Cleaning

During the library preparation, some contaminants could be introduced due to the non-perfect efficiency of the 'RNA enrichment' phase. SOLiD RNA-seqs were cleaned by contaminants using an implementation of PASS program that removes all sequences that map onto a collection of contaminants. The contaminants resulted less than 1% of the total sequences in all analyzed samples.

The collection of contaminants includes:

(1) UniVec (Cochrane et al.) (ftp://ftp.ncbi.nih.gov/pub/UniVec/)
(2) Database of mitochondrial genomes
    (ftp://ftp.ncbi.nlm.nih.gov/genomes/MITOCHONDRIA/Metazoa)
(3) Collection of E. Coli strain genomes.
(4) GtRNAdb a database of transfer RNA genes detected in genomic sequence
    (http://gtrnadb.ucsc.edu/) .
(5) RDP database (http://rdp.cme.msu.edu/seqmatch/seqmatch_intro.jsp).

De novo assembling

The version of *SATRAP* pipeline was 0.1, while Velvet assembly 1.2.10 and Oases 0.2.8.

The *SATRAP* pipeline run with the following parameters:

```
bin/satrap -step 1 2 3 4 \
-kmer_set 31 29 27 25 \
-reads_path SAMPLE_DIR/  \
-file_esten .csfastq \
-velvet_path  velvet_1.2.10_path/  \
-oases_path oases_0.2.8_path/ \
-q 28 -t1 5 -t2 0
```

For details about setting please, see the SATRAP manual at http://satrap.cribi.unipd.it .

STATISTICS

**Developmental Phase**	**N50**	**Size (bases)**	**Contig number**
TO	549	57,459,032	149067
MC	562	64,451,533	166584
Pre-TO	515	65,460,907	176885

Table S1: De novo transcriptome assembly statistics obtained using the SATRAP pipeline.
