# Supplementary material for: Transcriptome dynamics in the asexual cycle of the chordate Botryllus schlosseri
Source: BMC Genomics. 2016 Apr 2;17:275. doi: 10.1186/s12864-016-2598-1 (PMC4818882; doi:10.1186/s12864-016-2598-1)
Supplement: Additional file 2: — Genomic approach. Setting of programs used for genomic approach. (PDF 80 kb) [file 12864_2016_2598_MOESM2_ESM.pdf]

Genomic Approach

Mapping

The Solid sequenced reads were mapped using the program PASS with the following parameters:

Global alignment

```
pass -csfastq reads_file.csfastq \
-d reference_genome.fasta \
-fid 90  -fle 30 -p 11111111111111 \
-g 0 -cpu 12 -sam -b -flc 4 \
-seeds_step 8 -check_block 5000 -block 100000 \
 -query_size 1000 -auto_seeds_limit 30 \
-not_aligned  not_aligned_reads_because_spliced.csfastq
> sample_output.sam
```

Local alignments

```
pass -csfastq not_aligned_reads_because_spliced.csfastq \
-d reference_genome.fasta \
-query_size 100 -cpu 12 -fid 90 -original \
-p 11111111111111  -sam -max_trim_len \
 35 -flc 1 -seeds_step 1 -l -fle 12 -repeat -b \
> new_csfastq_with_mapped coordinates.csfastq
```

Spliced alignments

```
pass -csfastq new_csfastq_with_mapped coordinates.csfastq \
-query_size 100 -cpu 12 -fid 90 -spliced rna \
-e 0.5  -focus -max_distance 30000 \
-percent_tolerance 30 -no_trim_auto \
 -original  -p 111111111111 -sam  -flc 1 \
-seeds_step 1 -b -fle 10 -repeat -max_trim_len 35 \
>> sample_output.sam
```

Pairing

```
pass \
-sam1 first_sequenced_end.sam  \
-sam2 second_sequenced_end.sam  \
-pe_type 0 -range 0 600 1000  \
 -unique_pair 1 -unique_single 1 \
-not_unique_pair 1  -cpu 12 -stdout -ram \
> paired-alignments.sam
```

Mapped reads were filtered as follows: any read with a sequence identity less than 90% was filtered; any read, which had less than 30 bases in length, was filtered; any paired-end, for which only one end had mapped to produce multiple alignments, was filtered. The total mapped reads for each library are detailed in Table S2

Sample	Unique paired-end Mapped	Non unique paired-end Mapped	Unique single ends Mapped	Non unique single ends Mapped
**1186-R1**	4900816	7458623	8811929	18254861
**1186-R2**	5787389	4140349	14259378	15001592
**1186-R3**	4237284	6791517	8209262	18241781
**1186-R4**	6824169	9835321	12910683	25496540
**1186-R5**	4236263	6706500	9056668	19903279
**982-R1**	2821182	5193653	6361970	15605432
**982-R2**	3971093	4867662	9256387	16925513
**982-R3**	3880213	6582014	8132431	19417555
**982-R4**	4015404	5719757	9105323	18519508
**982-R5**	5460062	8757167	11518283	27151324
**985-R1**	5776838	9780749	12744413	30245214
**985-R2**	3903193	4352457	9827236	15658676
**985-R3**	3895936	5614507	8243480	16498591
**985-R4**	5023271	7554746	11733104	24303126
**985-R5**	6262693	9501482	16751981	40119229

Table S2: Align then assembly approach mapping information. We have considered 3 developmental phases indicated as 1186 (TO), 982 (MC) and 985 (pre-TO) listed in the column 'Sample'. The suffix -R followed by a number is associated to each biological replica. The columns 'unique paired-ends', 'non unique paired-ends', 'unique single-ends' and 'non unique single-ends' represent the number of unique and not unique alignments that could be paired or not.

Removing Clonal reads

Pair-ends were collapsed to remove clonal reads potentially produced in the PCR amplification from the same template molecules. Clonal sequences was removed using the program 'sam_clonality' from the PASS package with the following parameters:

```
sam_clonality  -sam sample_output_ordered.sam  -pe_overlap -multimap
```

where:

-sample_output_ordered.sam	The file contains the aligned reads ordered by their position for each chromosome of the reference genome.
-pe_overlap	It doesn't merge the overlapped paired-end alignments.
-multimap	It considers also multimap alignments.

See the manual of PASS at http://pass.cribi.unipd.it for further details about setting.
