# Supplementary material for: Transcriptome dynamics in the asexual cycle of the chordate Botryllus schlosseri
Source: BMC Genomics. 2016 Apr 2;17:275. doi: 10.1186/s12864-016-2598-1 (PMC4818882; doi:10.1186/s12864-016-2598-1)
Supplement: Additional file 3: — Gene prediction based on mapped transcripts. Setting of programs used for the gene prediction. (PDF 76 kb) [file 12864_2016_2598_MOESM3_ESM.pdf]

Gene prediction based on mapped transcripts

CUFFLINKS data integration

A parsimonious set of transcripts was assembled from the RNA-seq data using the program CUFFLINKS. The input data consists of: i) the RNA-seq data mapped onto the reference genome (SAM format), ii) the genome annotation in GFF3 format. The version of CUFFLINKS program was 2.2.1.

Parameters used for CUFFLINKS analysis:

```
cufflinks-2.2.1.Linux_x86_64/cufflinks \
-o OUTPUT_DIRECTORY/ \
-g botznik-reference-genome.gff3 \
-u \
--library-type fr-secondstrand \
-I 20000 \
-j 0.20 \
-p 12 \
-L 2ndA \
sample1.sam sample2.sam …..
```

For details about the used parameters, please see the *CUFFLINKS* User Manual at: http://cufflinks.cbcb.umd.edu/manual.html

PASA

The gene prediction analysis was performed using the PASA program. The input data consists of: i) the reference genome (botznik-reference-genome.fa), ii) the transcript sequences obtained from '*De novo*' transcriptome assembly (TRANSCRIPTS.fa), iii) the gtf file containing a parsimonious set of transcripts resulted by the data integration analysis (transcripts.gtf) performed with CUFFLINKS program.

Parameters used for PASA analysis

```
Launch_PASA_pipeline.pl -C -c standard-config-file  \
-R -g botznik-reference-genome .fa --CPU 12  --ALIGNERS gmap \
-t TRANSCRIPTS .fa --TDN denovo.accs \
 --cufflinks_gtf transcripts.gtf  \
--transcribed_is_aligned_orient  \
>> PASA.txt  \
2>> PASA.log
```

For details about the used parameters, please see the *PASA* User Manual at:
http://pasapipeline.github.io/
