# Supplementary material for: Transcriptome dynamics in the asexual cycle of the chordate Botryllus schlosseri
Source: BMC Genomics. 2016 Apr 2;17:275. doi: 10.1186/s12864-016-2598-1 (PMC4818882; doi:10.1186/s12864-016-2598-1)
Supplement: Additional file 4: — Unmapped transcripts recovering. Method description and statistics for unmapped transcripts recovering. (PDF 109 kb) [file 12864_2016_2598_MOESM4_ESM.pdf]

Unmapped transcripts recovering

By default, PASA program considered only the transcripts with a percentage of mapped sequence >= 90%. Transcripts that had less than 10% of covered sequence were recovered for further analysis.
Basing on this criteria, 3262 transcripts were analyzed to identify which ones had a significant coding potential. The program Transdecoder (from the Trinity package) was used for this purpose.
In order to avoid the inclusion of putative chimeric assemblies as well as possible, only the class "Complete ORFs" was analyzed (581 transcripts).
Those ORFs were traduced into amino-acidic sequences and then aligned onto the nr database using the BLASTP program. The aligned proteins were considered to calculate the percentage of transcripts sequence covered by the best hit protein (see Figure 1 below). Basing on this data we also inferred the CDS taxonomy of unmapped transcripts (figure 4 in the main article).

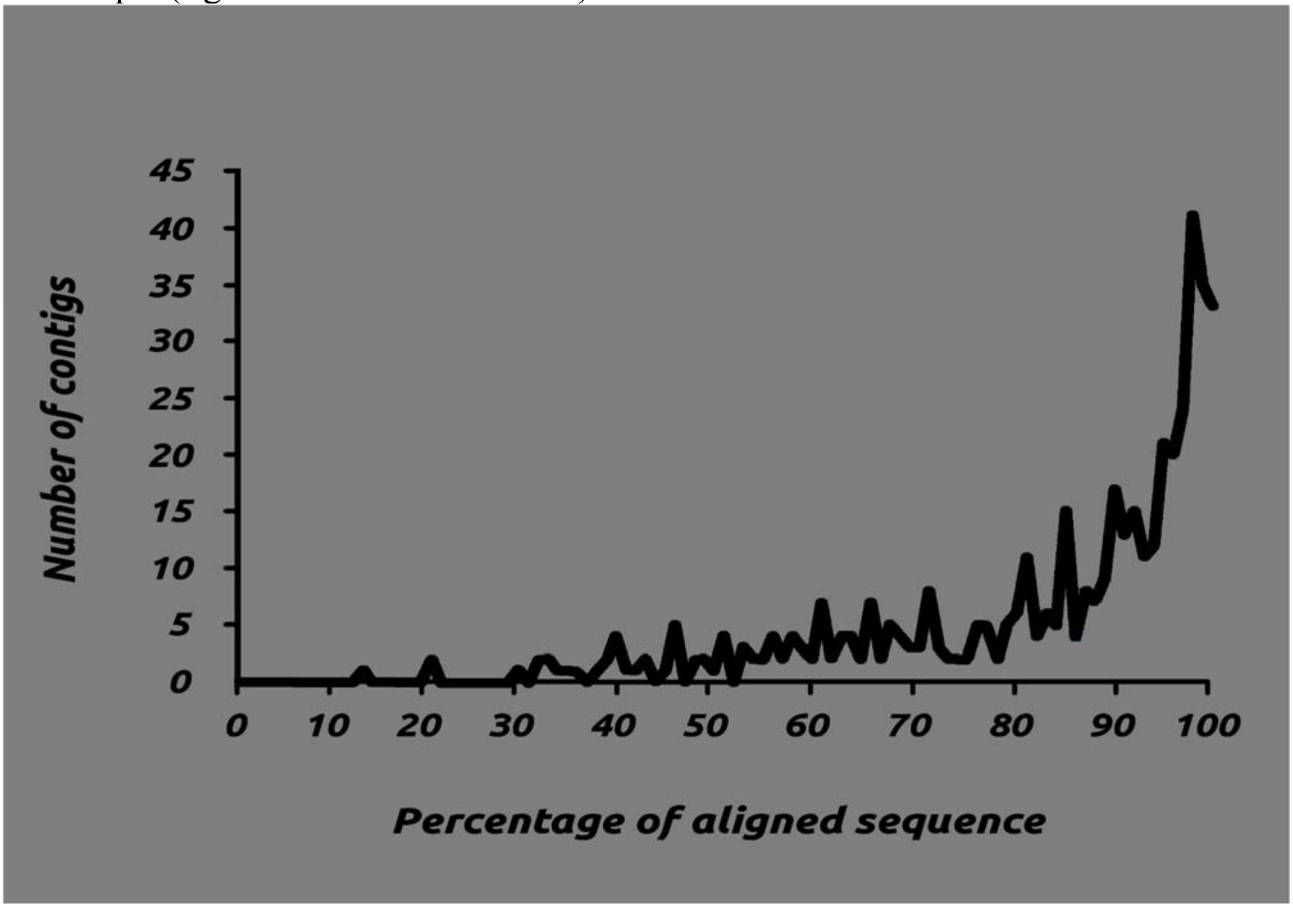

Figure 1. Percentage of transcript sequence length covered by the best hit protein. In this analysis we considered only the significant alignments (e-value <= 1**E-4)**. The number of ORFs that match only onto the best hit protein were 453 (78%) and 266 out off 581 (59%) mapped for more than 90% of their sequence length.
